# Viral vectored transmission blocking vaccines against *Plasmodium falciparum*

**DOI:** 10.1186/1475-2875-9-S2-O22

**Published:** 2010-10-20

**Authors:** Melissa C Kapulu, Sumi Biswas, Andrew Blagborough, Sarah C Gilbert, Robert E Sinden, Adrian VS Hill

**Affiliations:** 1 Jenner Institute, University of Oxford, Roosevelt Drive, Oxford, OX3 7DQ, UK; 2 Division of Cell and Molecular Biology, Sir Alexander Fleming Building, Imperia College London, Imperial College Road, London, SW7 2AZ, UK

## Background

Transmission blocking vaccines (TBVs) target sexual develop¬ment of the parasite within the mosquito and aim to prevent transmission of malaria from one individual to another. Antibodies raised against Pfs48/45, Pfs230 Region C, PfHAP2, and *Anopheles gambiae* Alanyl Aminopeptidase N1 (AgAPN1) proteins reduce transmission i.e. have transmission blocking activity [1-5]. Recombinant simian Adenovirus (AdC63 serotype) and Modified Vaccinia Ankara (MVA) viral vectors have been shown to induce high antibody titres to asexual parasite antigens in animal studies [6].

## Materials and methods

Protein sequences for each of the antigens were codon optimised for expression in humans and cloned into shuttle vectors, which were then recombined with the parental virus and purified to obtain virus expressing the antigen of interest. Mice were vaccinated with AdC63 (i.m.), sera was taken after 2 weeks, and will be followed by an MVA boost (i.d.) eight weeks after the prime. Antibodies were assayed by a standardised ELISA, and transmission blocking activity assessed using a standardised membrane feeding assay (SMFA).

## Conclusion

Induction of high antibody tires using this vaccine platform could be used together with other control measures to achieve elimination and/or eradication of the disease at a local or national level.
